# Assessment of Food Insecurity, Diet Quality, and Mental Health Status Among Syrian Refugee Mothers with Young Children

**DOI:** 10.3390/healthcare13233083

**Published:** 2025-11-27

**Authors:** Sedat Coşkunsu, Müge Yılmaz

**Affiliations:** 1 Department of Nutrition and Dietetics, Erciyes University Institute of Health Sciences, Kayseri 38280, Turkey; sedatcoskunsu@artuklu.edu.tr; 2 Department of Nutrition and Dietetics, Faculty of Health Sciences, Mardin Artuklu University, Mardin 47200, Turkey

**Keywords:** food insecurity, Syrian refugee, mental health, diet quality

## Abstract

**Background:** Although Türkiye hosts the largest population of Syrian refugees in the world, research on the vulnerability factors contributing to food insecurity among Syrian refugee mothers remains scarce. The aim of this study was to investigate the relationships between maternal food insecurity, diet quality, and mental health outcomes among Syrian refugee mothers with young children living in Türkiye. **Methods:** This cross-sectional study involved 285 Syrian mothers living in Türkiye with children under five years of age. Maternal food insecurity was assessed via the Food Insecurity Experience Scale (FIES), diet quality was evaluated via the Healthy Eating Index-2020 (HEI-2020), and the Patient Health Questionnaire-9 (PHQ-9) was used to evaluate mental health conditions. Data were collected through face-to-face surveys conducted by two native Arabic translators, and the analyses included sociodemographic characteristics, anthropometric measurements, diet quality, food insecurity status, and mental health status. **Results:** The prevalence of moderate/severe food insecurity and poor diet quality among refugee mothers amounted to 30% and 59.3%, respectively. Mothers experiencing food insecurity presented significantly lower levels of mental health and diet quality (*p* < 0.05). A one-unit increase in the food insecurity score was associated with an increase of 1.031 units in the total Patient Health Questionnaire-9 (PHQ-9) score. The model demonstrated that food insecurity accounted for 30.2% of the variance in PHQ scores (R^2^ = 0.302). Low income, lack of institutional aid, short length of stay, and number of children significantly increase the likelihood of poor dietary quality among refugee mothers. **Conclusions:** Food insecurity was found to be a widespread problem for mothers who are refugees from Syria. It was significantly associated with poorer nutritional quality and mental health issues in mothers. These findings suggest the need for expanding social support programs, implementing effective public health interventions for food security, and securing an overall improvement of maternal health.

## 1. Introduction

Food insecurity refers to the inability to secure enough nutritionally adequate food with which to maintain a healthy and active life [1]. Food insecurity, a global challenge estimated to affect 30% of the worldwide population, is further compounded among refugees who are exposed to heightened vulnerability after conflict-related instability results in displacement [2]. According to the 2024 United Nations report on the State of Food Security and Nutrition, an estimated 2.33 billion people faced moderate or severe food insecurity in 2023 [3].

Food insecurity (FI) is a critical public health and socioeconomic issue affecting vulnerable populations, particularly refugees and those living in poverty [4]. It arises from multiple interrelated factors, such as limited income, unemployment, educational disadvantages, and restricted access to essential services [5,6]. The literature shows that lower socioeconomic status, living in a larger household, and limited educational attainment are significant predictors of FI [7,8,9]. Refugees constitute one of the most at-risk groups due to their precarious living conditions and the traumatic experiences associated with displacement and resettlement [10,11,12]. This situation exacerbates their nutritional vulnerability, often resulting in poor diet quality, low dietary diversity, and higher rates of micronutrient deficiencies, especially in female-headed households [12,13,14].

The relationship between food insecurity and mental health is well established [13,15] and has been consistently reported among Syrian communities residing in various host countries, including Lebanon, Jordan, Iraq, Türkiye, and the United States [16,17,18,19]. The protracted nature of the ongoing conflict in Syria and its multifaceted consequences have heightened Syrians’ vulnerability to poor mental health outcomes. This susceptibility is primarily linked to the exacerbation of pre-existing mental health disorders, the emergence of new psychological concerns, and challenges in adapting to unstable living conditions [20]. Moreover, previous research has demonstrated that there is a significant association between food insecurity and depressive symptoms [21]. Refugees are often exposed to numerous displacement-related stressors, such as loss, uncertainty, and social isolation, which further increase the risk of developing mental health problems [22]. In this vein, a study demonstrated that refugees account for a greater percentage of psychological disorders than the general population [23].

The Syrian civil war has led to one of the largest refugee crises ever, with millions displaced and relocated to neighboring countries and other countries around the world. Hosting 2.7 million refugees from Syria, Turkey is the country that hosts the largest Syrian refugee population globally [24]. The country adopted a policy of temporary protection to help Syrians enter the country and to offer them medical care, social support, psychological care, and education [25]. The government has introduced comprehensive aid programs, but studies indicate that food insecurity is common among Syrian refugees with poor nutritional and mental health statuses [26,27,28].

According to the literature, food insecurity is associated with poor physical and mental statuses among adults and children, especially females [29,30]. Mothers are found to adopt coping strategies such as restricting and skipping meals in food-insecure homes to prevent their children from hunger, leading to poor nutritional quality and posing health threats [30,31]. There are few studies related to food insecurity among Syrian refugees resettled in Turkey [26,32]. It is vital to conduct studies based on genuine scientific data because proper policies and strategies are formulated accordingly and tailored specifically to the needs of the refugee population [33]. In this study, we aimed to investigate the associations between food insecurity, diet quality, and mental status, as well as the sociodemographic factors that may influence these relationships, among Syrian refugee mothers of young children in Türkiye.

## 2. Materials and Methods

### 2.1. Study Design and Population

This cross-sectional study was carried out with Syrian mothers who applied to the Migrant Health Center in Mardin province between May and August 2024. This study was approved by the ethics committee of Mardin Artuklu University (decision number: 2024/5-18).

A two-stage purposive sampling approach was used to determine the working group. In the first stage, the Mardin Migrant Health Centre, located in southeastern Turkey, was selected, as it serves a large Syrian refugee population and provides maternal and child health services. In the second stage, Syrian mothers who visited the center during the data collection period were deemed by healthcare workers to meet the inclusion criteria for the study and invited to participate. The province of Mardin in Turkey borders Syria and is an area containing a high concentration of Syrian refugees (46,097 registered refugees, constituting approximately 5% of Mardin’s total population) [34]. The province lies between 36°55′–37°30′ N latitudes and 40°30′–41°45′ E longitudes, bordering Şanlıurfa to the west, Batman to the north, Siirt to the northeast, and Şırnak to the east while sharing a southern border with Syria [35]. The inclusion criterion was being a Syrian mother aged 18–50 years with a minimum of one child below the age of 5. Pregnant mothers were excluded from this study. The sample size was calculated based on data from a prior study that estimated the degree of food insecurity among Syrian refugee women in Turkey to be 36% [32]. This calculation was performed via the program Epi Info 7.2. Population size (N = 1320) expected food insecurity prevalence (36%), the confidence level (95%), the margin of error (±5%), and the minimum required sample size (280) were considered. The study was determined to require a minimum of 280 participants. Of the 460 women initially approached, 375 Syrian mothers with at least one child under five years of age were eligible for inclusion. Among these eligible mothers, 310 (82.9%) provided consent to participate in the survey. Ultimately, a total of 285 mothers (91.9%) completed the interview and were included in this study.

### 2.2. Data Collection

The data for this study were gathered with the help of a structured questionnaire and face-to-face survey. Two native Arabic interpreters were employed to communicate properly with the mothers. The questionnaire was divided into four sections: (a) sociodemographic, (b) mental health status, (c) food insecurity status, and (d) anthropometric measurements and quality of diet.

The PHQ-9 is a nine-item scale based on the DSM-IV depression criteria. The PHQ-9 is also employed internationally for screening for depression among refugees and is valid and reliable [36,37]. The PHQ-9 scale was employed for the assessment of the severity of depressive symptoms, with a 0–27 point range (Cronbach’s α = 0.84). The results of the scale were categorized as follows: minimal depression (1–4 points), mild depression (5–9 points), moderate depression (10–14 points), moderately severe depression (15–19 points), and severe depression (20–27 points) [38].

Food insecurity was assessed with the Food Insecurity Experience Scale (FIES), a validated experience-based instrument developed by the Food and Agriculture Organization (FAO). The scale has good reliability and internal consistency (Cronbach’s α = 0.759) [39]. This eight-item scale assesses the experiences of people with respect to food security within the last 12 months. In this study, the participants were also asked whether they had ever worried about not having enough food, used all the food they had at home, or reduced the quality or quantity of food they consumed because they did not have enough money or food. Each affirmative response (“yes”) was assigned 1 point, whereas negative responses (“no”) were assigned 0 points. A total score ranging from 0 to 8 was obtained by summing the scores from all eight items. The respondents’ food insecurity statuses were categorized according to the total score, as follows: food security (score = 0), mild food insecurity (score = 1–3), moderate food insecurity (score = 4–6), and severe food insecurity (score = 7–8) [40,41]. Subsequently, items 1, 2, 3, 4, 5, 6, 7, and 8 were combined to obtain a single variable. This variable classified households with a score of 4 or higher (≥4) as having moderate-to-severe Food Insecurity (FI) and those with a score between 0 and 3 as having no or low Food Insecurity (FI) [42].

### 2.3. Anthropometric Measurements and Diet Quality

Mothers’ weight, height, and waist circumference were measured according to the method reported in [43,44]. Maternal nutritional status was determined via a multifaceted anthropometric assessment. The following parameters were employed in this assessment: body mass index (BMI), waist circumference, and waist-hip ratio. These measurements were interpreted according to the guidelines established by the World Health Organization (WHO). Body mass index (BMI), calculated as weight (kg) divided by height squared (m^2^), was used to categorize the participants into the following groups: underweight (BMI < 18.5 kg/m^2^), normal weight (18.5–24.9 kg/m^2^), overweight (25.0–29.9 kg/m^2^), and obese (≥30.0 kg/m^2^). Waist circumference was used to evaluate the risk for metabolism, with measurements >80 cm and >88 cm representing increased and high risk, respectively. A waist-to-hip ratio ≥0.85 was also identified as a primary marker for a health risk factor [45,46]. Anemia status was determined based on clinical records obtained from local health centers. The participants were categorized as either “anemic” or “non-anemic” according to prior medical diagnoses recorded by healthcare professionals. Anemia was defined as hemoglobin levels (Hb < 120 g/dL) according to the WHO criteria [47]. A specialist physician performed the evaluation. The portion sizes and quantities of food were obtained by using the ‘Food and Food Photo Catalogue: Measurements and Quantities’ as a reference point [48]. The quality of the mothers’ diets was evaluated via the Healthy Eating Index-2020 (HEI-2020), which was first established in 2005 and has been updated periodically thereafter based on the American Dietary Guidelines. The 24 h recalls were used to assess diet quality. A single 24 h dietary recall was administered for each participant on a randomly selected weekday. The updated HEI-2020 comprises 13 distinct components, encompassing the evaluation of food diversity and nutrients. Utilizing the data obtained from the participants’ food consumption records, we scored and evaluated the nutrients and nutrient content consumed by individuals in each 1000 kcal according to the reference consumption amounts. The range of scores achievable on the questionnaire is between 0 and 100. According to the HEI-2020, a score of ≤50 is defined as ‘poor diet quality’, a score of 51–80 is defined as ‘needs improvement’, and a score >80 is defined as ‘good diet quality’ [49,50].

### 2.4. Ethics

Ethical approval was obtained from the Mardin Artuklu University Non-Invasive Clinical Research Ethics Committee (Date: 7 May 2024; Reference number: 2024/5-18). Written informed consent was obtained from each participant included in this study. This study was conducted in accordance with the principles of the Declaration of Helsinki.

### 2.5. Statistical Analysis

Statistical Package for Social Sciences (SPSS) version 25 was used for data analysis. Based on FIES scores, the participants were classified as food-secure (including food-secure and mildly food-insecure women) or food-insecure (including moderately and severely food-insecure women) for analysis. Categorical variables were presented as frequencies and percentages, and group comparisons were performed using the chi-square test. Continuous quantitative data are expressed as means and standard deviations (X ± SD). The Kolmogorov-Smirnov test was used to assess the assumption of a normal distribution of the quantitative data. An independent *t*-test was used to compare means between two groups for normally distributed parameters. Hierarchical regression and binary logistic regression methods were employed in multivariate analyses. The goodness of fit of the logistic regression model was assessed via the Hosmer–Lemeshow test. All the models were adjusted for age, maternal education level, food insecurity, income status, number of children, and duration of stay. Statistical significance was considered *p* < 0.05 for all analyses.

## 3. Results

**Figure 1:**
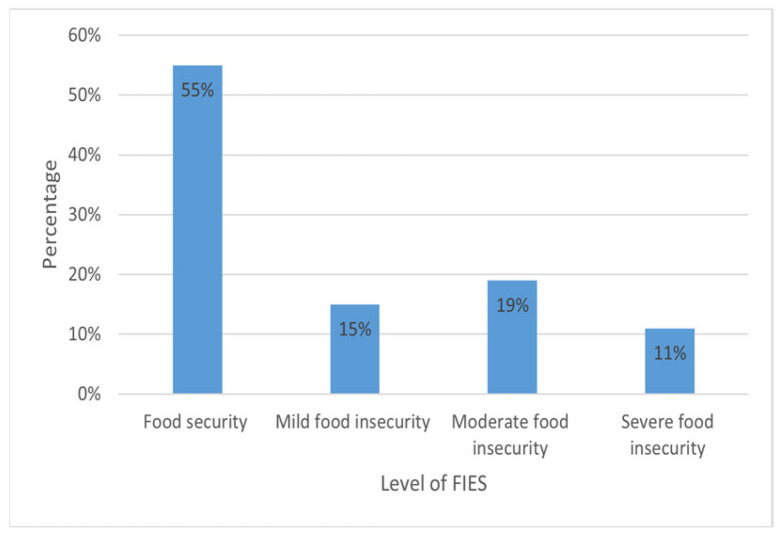
Levels of food insecurity among adult women Syrian refugees.

**Figure 2:**
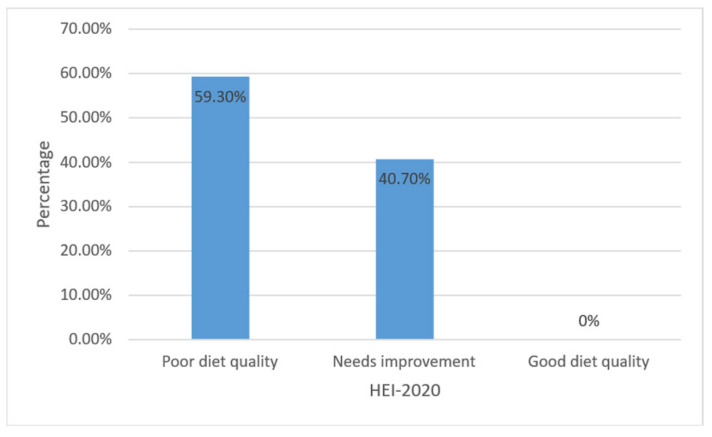
HEI-2020 scores for Syrian refugee women.

A total of 285 mothers were included in this study. The mean age of the mothers was 29.28 ± 6.23 years, their mean BMI was 26.27 ± 5.46, and approximately half of the mothers (54%) had attained primary school education. The mean number of children in each household was 3.09 ± 1.46, and the mean duration of living in Türkiye was 9.27 ± 2.21 years. The majority of the mothers were housewives (99.6%). The prevalences of poor diet quality, mild depression, and food insecurity in the study population were 59.3%, 35.8%, and 45%, respectively. Overall, 55% of the women were food secure, while 45% experienced food insecurity, with 15%, 19%, and 11% experiencing mild, moderate, and severe levels of food insecurity, respectively (Figure 1). In regard to diet quality evaluation based on the HEI-2020 scores, approximately 59.3% were categorized as ‘poor’, while 40.7% were categorized as ‘needs improvement;’ none (0%) of the patients were deemed to have good diet quality (Figure 2).

Significant associations were found between most of the demographic variables and food insecurity status. The relationships between all the demographic and nutritional variables according to food security status are presented in Table 1. The mental health statuses of individuals affected by food insecurity (13.74 *±* 5.34) was determined to be statistically significantly higher relative to those with food security (9.88 *±* 4.37) (*p* < 0.001). Furthermore, the diet quality score was found to be statistically significantly higher for mothers with food security (50.98 *±* 11.43) than those suffering from food insecurity (47.89 *±* 8.88) (*p* = 0.014). However, no significant difference in anthropometric measurements was observed based on food security status. A comparison of maternal education levels showed that there was a significantly higher proportion of participants with illiterate mothers in the food-insecure group (*p* = 0.001). The distribution of the duration of residence in Türkiye also differed significantly between the groups (*p* < 0.001). The vast majority of women in the food-insecure group had resided in Türkiye for 5–7 years, whereas the majority of the food-secure group had resided in the country for 8–10 years. Regarding anemia status, there were more anemic women in the food-insecure than in the food-secure group; this difference was statistically significant (*p* = 0.007).

Table 2 presents the results of the hierarchical regression model analysis. When the FIES score was added to the first model (F = 123.833), the findings indicated that food insecurity explained 30% of the variance in the PHQ-9 scores (R^2^ = 0.302). Once income status was added to the model, the second model explained 34% of the variance in PHQ-9 scores (R^2^ = 0.339), and the change in the coefficient of determination was found to be statistically significant (F = 73.840, *p* < 0.001). With the addition of the duration of stay in Turkey, the third model explained 36% of the variance in PHQ-9 scores, and the change in the coefficient of determination was again statistically significant (F = 55.988, *p* < 0.001). All the variables independently influenced and predicted PHQ-9 scores.

We examined the key socioeconomic and demographic determinants affecting the dietary quality of refugee mothers. The simple logistic regression analysis presented in Table 3 demonstrated a significant association between dietary quality and income status, receipt of aid from an organization, duration of stay, and number of children (*p* < 0.05). Findings obtained via a multiple logistic regression model revealed that mothers with low income were 1.71 times more likely to have poor dietary quality than mothers with higher income levels (adjusted OR = 1.71; 95% CI: 1.01–2.89). The likelihood of poor dietary quality was 3.15 times greater for mothers who did not receive aid from an organization than for those who did (adjusted OR = 3.15; 95% CI: 1.56–6.37). With an increase in the number of children, the risk of poor dietary quality also significantly increased (adjusted OR = 2.31; 95% CI: 1.10–4.81 and 5.90; 2.47–14.05). Interestingly, mothers with a shorter duration of stay had a greater risk of poor dietary quality than those with a longer duration of stay (adjusted OR = 6.75; 95% CI: 2.81–16.18 and 2.70; 1.48–4.91).

## 4. Discussion

This study examines the interrelationships between food insecurity, diet quality, and mental health outcomes among Syrian refugee mothers residing in Mardin, Türkiye. The findings reveal a high prevalence of food insecurity (30%), and mothers with higher levels of food insecurity had significantly lower diet quality and higher depressive symptom scores. The findings also show that less education, shorter duration of residence, lack of institutional assistance, and having a greater number of children were significantly associated with increased food insecurity. In addition, low income, absence of institutional support, shorter residence duration, and having a greater number of children were identified as significant determinants of poor diet quality.

The prevalence of moderate/severe food insecurity identified in this study (30%) is lower than that reported among Syrian refugees in Jordan (78%) and Lebanon (65%) but higher than that observed among resettled refugees in Norway (22%). Studies from Canada (52–77%) and the United States (80%) similarly indicate that food insecurity remains a substantial issue even in high-income settings [4,12,51,52,53]. These cross-national variations likely reflect differences in welfare systems, social protection coverage, and refugee integration policies. Similar patterns have also been observed across different regions of Türkiye. The World Food Programme (WFP) reported a food insecurity prevalence of 36% among Syrian refugees residing in Gaziantep, Hatay, Kilis, and Şanlıurfa, while another study conducted in Samsun found a prevalence of 47% [32,54]. In contrast, a much higher rate (90.3%) was reported among urban refugee populations in Istanbul, where the cost of living is substantially higher and access to formal employment remains limited [26]. These variations within Türkiye illustrate the influence of regional economic disparities, urban living costs, and employment opportunities on food security status.

Food insecurity is strongly shaped by socioeconomic determinants such as education, income, and social support systems, as highlighted in previous studies [54,55,56]. Evidence from refugee populations demonstrates that declining household income and limited access to employment opportunities exacerbate the risk of food insecurity [57,58]. In this study, we found no statistically significant differences in self-reported income status between food-secure and food-insecure Syrian refugee women, suggesting that the limited availability of formal employment for refugees and their unstable income sources may restrict the protective effects of income stability. Educational attainment emerged as another important determinant of food security. As with earlier findings obtained from migrant populations in Canada and Europe, in our sample, mothers with lower education levels reported significantly higher food insecurity [58,59]. This pattern reinforces the idea that education may enhance not only economic capacity but also food literacy and the ability to navigate host-country food systems. Furthermore, the association between a shorter duration of residence and higher food insecurity supports the “acculturation hypothesis,” which posits that newcomers initially face structural and linguistic barriers that gradually diminish with time [60]. Institutional assistance, whether through food aid or cash transfers, has been identified as a crucial buffer against food insecurity across various refugee settings [51,61]. Our findings show no significant differences in the prevalence of receiving food aid between the food-secure and food-insecure groups. This underscores the necessity of complementing in-kind or cash assistance with broader interventions that address the root causes of economic instability, such as access to formal labor markets and sustainable livelihoods, to more effectively ensure food security in refugee populations. Addressing food insecurity in displaced populations therefore requires a multidimensional approach that combines socioeconomic empowerment, education, and targeted humanitarian support.

Numerous studies have consistently demonstrated that there is a significant association between food insecurity and low dietary diversity among mothers [37,62,63]. These findings reflect how economic hardship limits access to nutrient-rich foods and increases reliance on inexpensive, energy-dense products. In line with studies involving refugee mothers in Lebanon, Nepal, and Malawi [37,62,63], we also found that food-insecure mothers had significantly poorer diet quality scores. This pattern underscores the fact that food insecurity not only affects the quantity of food consumed but also reduces dietary diversity and nutrient adequacy—key indicators of nutritional well-being.

Economic vulnerability and the high cost of nutrient-dense foods often drive households toward the consumption of low-cost staples rich in fats and sugars [64]. This adaptive behavior, while helping to meet energy needs, leads to nutritional imbalances and long-term health risks. Several studies have identified a “double burden of malnutrition” in such contexts, where micronutrient deficiencies coexist with overweight and obesity due to the predominance of energy-dense, nutrient-poor diets [65,66,67]. These findings are highly relevant for refugee populations, including those in Türkiye, who face financial insecurity, limited market access, and high food prices.

Moreover, this study reveals a significant association between food insecurity and anemia, corroborating evidence from other low- and middle-income countries [68,69]. This physiological link may stem from inadequate intake of iron-rich foods, lower dietary diversity, and greater consumption of refined grains and sugars in food-insecure households. Nonetheless, some studies have not found this relationship [37,70], possibly due to differences in the measurement of anemia, the inclusion of fortified foods, or the availability of humanitarian nutrition interventions. Taken together, these results suggest that while the pathways between food insecurity, poor diet quality, and micronutrient deficiency are complex, they converge on the same structural determinant—limited economic and social access to adequate nutrition

Refugees have been found to experience higher rates of mental health problems relative to the general population, driven by displacement-related stressors such as trauma, socioeconomic hardship, and social isolation [12,23]. In Türkiye, Acarturk et al. reported that nearly half of Syrian refugees living in Istanbul (46.9%) exhibited symptoms of at least one common mental disorder, and one-third had comorbid mental health conditions [27]. Similar trends have been documented among displaced populations in Lebanon, Uganda, and Australia, underscoring the global burden of psychological distress among refugees [23,71]. Food insecurity has emerged as a significant psychosocial stressor that exacerbates the risk of poor mental health outcomes [21,37,71]. Consistent with previous meta-analyses and regional studies, we found that mothers experiencing food insecurity have significantly higher depressive symptom scores. Specifically, a one-unit increase in food insecurity was associated with a 1.03-unit increase in mental disorder scores, confirming the strong positive correlation between nutritional and psychological vulnerability. The pathways linking food insecurity to mental health are multidimensional. Chronic worrying about food availability, social stigmas associated with poverty, and the daily struggle to secure sufficient nutrition contribute to sustained psychological distress. Moreover, inadequate dietary intake may directly influence neurobiological mechanisms through micronutrient deficiencies that impair mood regulation and cognitive functioning [21]. Refugee women, in particular, face a dual burden—managing household food insecurity while bearing caregiving and social adjustment responsibilities in a foreign environment—which amplifies stress and depressive symptoms [37].

Evidence from longitudinal and cross-sectional studies indicates that the risk of food insecurity among refugees is highest shortly after arrival and gradually decreases as they adapt to the host country’s socioeconomic environment [12,72]. A Norwegian study found that newly resettled refugees frequently experienced food insecurity due to limited income and social integration barriers, yet this rate declined over time as refugees improved their employment statuses and adapted to the local food environment [72]. Similarly, Kamelkova et al. reported that prolonged residence in Norway was associated with both reduced food insecurity and improved mental health outcomes, emphasizing the protective role of socioeconomic adaptation [12]. Economic hardship during the resettlement process imposes an additional psychological burden, particularly as refugees struggle to meet basic family needs such as food, housing, and healthcare. The convergence of migration-related trauma and post-migration financial stress can elevate anxiety, depression, and overall distress levels. These mechanisms are consistent with the “post-migration stress model,” which posits that economic and social stressors in the host country can sustain or even intensify psychological difficulties long after the migration event itself [73]. In this study, income level accounted for 34% of the variance in maternal mental health scores, while duration of stay in Türkiye explained 36%. This finding supports the adaptation hypothesis, suggesting that longer residence may mitigate psychological distress by increasing access to resources, employment, and social support networks. Nevertheless, persistent economic vulnerability and limited access to stable employment continue to place refugee mothers at elevated risk of both food insecurity and poor mental health.

Previous studies concerning Syrian refugee mothers showed that low income and household crowding are strongly linked to poor dietary quality [14,37]. Consistent with these findings, our study reveals that mothers with lower income and more children had significantly lower diet quality scores. Low-income families often rely on inexpensive, calorie-dense foods that meet energy needs but lack essential nutrients [64]. On the other hand, in this study, individuals receiving aid from an organization were found to have higher diet quality. Additionally, the association between improved diet quality and longer residence duration suggests that economic adaptation and greater familiarity with local food systems may facilitate better nutritional outcomes. However, as shown in a previous study [28], this relationship may vary depending on the resettlement stage and adaptation period.

Limitations of this study: First, the sample was limited to mothers who applied to a Primary Health Services Center in one province (Mardin), restricting the degree to which the findings can be generalized to all Syrian refugees at the national level, particularly those in rural or camp settings. Moreover, most of the participants were stay-at-home mothers, so the sample may not represent the broader refugee population. Second, dietary intake was assessed using a single 24 h recall, which might not fully represent habitual dietary patterns. Future studies should incorporate repeated recalls or food frequency questionnaires to capture long-term dietary behaviors. Third, data were collected between May and August, a period that does not reflect potential seasonal variations in food availability, market prices, or dietary habits. Lastly, this study’s cross-sectional design precludes causal inference; longitudinal research must be conducted to clarify the directionality of the relationships between food insecurity, diet quality, and mental health.

## 5. Conclusions

This study reveals the prevalence of FI, poor dietary quality, and mental issues among Syrian refugee mothers who live in an urban-crisis setting. The findings indicate that poor diet quality is prevalent within this population. Increased socioeconomic vulnerabilities and food insecurity have negative impacts on dietary quality and mental status. To address this complex issue, researchers must adopt a holistic approach using interventions with multiple dimensions—such as nutrition-focused interventions and aid schemes, which could be developed at the national and local levels—or improve existing schemes. Additionally, psychosocial support and family planning services should be leveraged. To reduce food insecurity and promote diet quality in vulnerable populations, it is necessary to consider mothers’ roles as providers and caregivers. Further comprehensive studies are needed to explore the long-term impacts of the food insecurity of refugee mothers on diet quality and overall health status. Specifically, further examination of the complex interactions between mothers’ poor mental statuses, food insecurity, and mothers’ nutrition within the context of Syrian refugee mothers living in Turkey is necessary. To improve the nutritional statuses and mental well-being of mothers, it is crucial to develop and implement policy interventions based on scientific evidence at both the national and international levels.

## Figures and Tables

**Table 1:** Association between sociodemographic and nutritional variables according to food security status.

Variables	Food Secure (*n* = 198)	Food Insecure (*n*: 87)	*p*
Age (year)	29.20 *±* 6.11	29.48 *±* 6.54	0.727
HSA score (X ± SD)	9.88 *±* 4.37	13.74 *±* 5.34	<0.001 *
HEI score (X ± SD)	50.98 *±* 11.43	47.89 *±* 8.88	0.014 *
BMI (kg/m^2^) (X ± SD)	26.04 *±* 5.75	26.54 *±* 50.8	0.927
Waist circumference (cm) (X ± SD)	82.48 *±* 9.38	83.17 *±* 10.57	0.600
Waist/Hip (X ± SD )	0.87 *±* 0.04	0.88 *±* 0.05	0.102
Mother’s education level			
Illiterate	12 (5.7%)	15 (20.0%)	0.001 **
Literate	38 (18.1%)	12 (16.0%)	
Primary school	115 (54.8%)	40 (53.3%)	
High school	45 (21.4%)	8 (10.7%)	
Duration of stay (years)			
5–7 years	20 (9.5%)	36 (48.0%)	<0.001 **
8–10 years	120 (57.1%)	26 (34.7%)	
11–13 years	70 (33.3%)	13 (17.3%)	
Number of children			
1–2	75 (35.7%)	27 (36.0%)	0.828
3–4	80 (38.1%)	26 (34.7%)	
≥5	55 (26.2%)	22 (29.3%)	
Income status			
Equals expenses	95 (45.2%)	32 (42.7%)	0.803
Less than expenses	115 (54.8%)	43 (57.3%)	
Receiving food assistance			
Yes	125 (59.5%)	37 (49.3%)	0.163
No	85 (40.5%)	38 (50.7%)	
Anemia status			
Anemic	60 (28.6%)	35 (46.7%)	0.007 **
Not anemic	150 (71.4%)	40 (53.3%)	

Significantly different at *p*-value < 0.05. * Independent samples *t*-test. ** Chi-squared test.

**Table 2:** Hierarchical regression model of the impact of maternal food insecurity, income status, and duration of residency in the host country on mental health scores.

Model		Food Insecure Score	İncome Status	Time Duration	*R^2^*
Model 1	B	1.031			0.302
	SE	0.093			
	*β*	0.552			
	t	11.128 *			
Model 2	B	1	2.075		0.339
	SE	0.09	0.505		
	*β*	0.535	0.199		
	t	11.055 *	4.110 *		
Model 3	B	0.729	2.098	−0.531	0.367
	SE	0.115	0.494	0.144	
	*β*	0.39	0.201	−0.227	
	t	6.344 *	4.246 *	−3.695 *	

t *; *p* < 0.05. All the models were adjusted for age, maternal education level, and number of children.

**Table 3:** Multivariable binary logistic regression regarding key components determined by maternal diet quality and its associations.

Variable	Needs Improvement	Poor Diet	OR (95% CI)	p *	aOR (95% CI)	p **
	(*n*: 116)	(*n*: 169)				
Mother’s education						
Illiterate	10 (8.6)	17 (10.1)	1		1	
Literate	19 (16.4)	31 (18.3)	0.96 (0.36–2.52)	0.934	1.78 (0.60–5.25)	0.299
Primary school	60 (51.7)	95 (56.2)	0.93 (0.40–2.16)	0.869	1.99 (0.76–5.24)	0.160
High school	27 (23.3)	26 (15.4)	0.56 (0.21–1.46)	0.24	1.45 (0.48–4.35)	0.501
İncome status						
Equal	61 (52.6)	66 (39.1)	1		1	
Low	55 (47.4)	103 (60.9)	1.73 (1.07–2.79)	0.024	1.71 (1.01–2.89)	0.048
Receiving food assistance						
Yes						
No	76 (65.5)	86 (50.9)	1	0.015	1	0.001
	40 (34.5)	83 (49.1)	1.83 (1.12–2.98)		3.15 (1.56–6.37)	
Time spent living in Türkiye						
5–7 years	10 (8.6)	46 (27.2)	8.12 (3.58–18.40)	<0.001	6.75 (2.81–16.18)	<0.001
8–10 years	53 (45.7)	93 (55.0)	3.10 (1.77–5.43)	<0.001	2.70 (1.48–4.91)	0.001
11–13 years	53 (45.7)	30 (17.8)	1		1	
Number of children						
1–2	49 (42.2)	53 (31.4)	1		1	
3–4	48 (41.4)	58 (34.3)	1.12 (0.65–1.93)	0.69	2.31 (1.10–4.81)	0.026
≥5	19 (16.4)	58 (34.3)	2.82 (1.48–5.39)	0.02	5.90 (2.47–14.05)	0.001

* OR of the dependent variable (poor diet vs. diet that needs improvement) are presented with 95% CIs determined using simple logistic regression. ** Adjusted OR (aOR) is presented with 95% CIs determined using multiple logistic regression analysis. The values are adjusted for age of the mother; marital status, education, and employment status of the parents; time spent living in the country; income status; number of children; and receipt of food assistance.

## Data Availability

The raw data supporting the conclusions of this article will be made available by the authors upon request due to privacy and ethical restrictions, in accordance with institutional ethical approval.

## Abbreviations

BMI	Body Mass Index
FIES	Food Insecurity Experience Scale
PHQ-9	Patient Health Questionnaire-9
HEI-2020	Healthy-Eating Index-2020
